# Editorial: Acute myeloid leukemia (AML): Is it time for MRD-driven treatment?

**DOI:** 10.3389/fonc.2022.1020185

**Published:** 2022-09-09

**Authors:** Fabio Guolo, Claudio Cerchione, Chiara Vernarecci, Alessandro Isidori

**Affiliations:** ^1^ Clinic Of Hematology, Department of Internal Medicine (DiMI), University of Genoa, Genoa, Italy; ^2^ Dipartimento di Oncologia ed Ematologia, IRCCS Ospedale Policlinico San Martino, Genoa, Italy; ^3^ Hematology Unit, IRCCS Istituto Scientifico Romagnolo per lo Studio e la Cura dei Tumori (IRST), Meldola, Italy; ^4^ Haematology and Stem Cell Transplant Center, Azienda Ospedaliera Ospedali Riuniti Marche Nord (AORMN), Pesaro, Italy

**Keywords:** acute myeloid leukemia (AML), minimal residual disease (MRD), immunotherapy, allogeneic stem cell transplantation, leukemic stem cell (LSC)

An increasing number of genetic and epigenetic abnormalities have been shown to display prognostic value in acute myeloid leukemia (AML) (1). Risk stratification at diagnosis, as defined by European LeukemiaNet (ELN) risk score, is important in order to define prognosis for AML patients (1). However, minimal residual disease (MRD) assessment during the therapeutic course further refines baseline risk definition (2, 3). There is indeed strong evidence that the persistence of MRD after first line treatment is associated with an inferior outcome in AML, regardless of baseline risk assessment (4–6). Even in the setting of allogeneic stem cell transplantation (HSCT), the presence of MRD before transplant, detected by any method, is related with significantly shorter survival, as it is confirmed by recent meta-analysis (7).

Traditionally, multicolour flow cytometry (MFC) and real time PCR based techniques targeting recurrent specific molecular alterations were the most widely used tools for MRD assessment in AML. More recently, next-generation sequencing (NGS) has been introduced. Despite good sensitivity of those methods, there are still some pitfalls in MRD evaluation in AML, for example, mutations associated with nonmalignant clonal hematopoiesis or immunophenotypic abnormalities due to regenerating bone marrow can determine a “background noise” that may result in false positive tests (8, 9).

Furthermore, the optimal timepoints for MRD assessment still have to be defined (10–12). In this view, Liu et al. evaluated a combination of PCR and MFC in a large cohort of AML patients treated with conventional 3 + 7 induction, demonstrating that the integration of the two techniques depends on the specific AML subtype, whereas the post -consolidation time point provided the most significant prognostic information. It is possible however that if more intensive induction therapies are adopted, the optimal timepoint for MRD assessment may differ (13, 14).

Differently from acute lymphoblastic leukemia, where almost all therapeutic protocols are MRD driven, in AML, despite the relevant prognostic role of MRD, only a small minority of protocols incorporate MRD information for therapeutic decisions (15). Furthermore, in most AML subtypes, MRD follow-up is not standardized yet, and, for relapsing patients, salvage therapy is usually administered at the time of overt hematological relapse (1). On the contrary, MRD assessment is crucial for many relevant clinical decisions in acute promyelocytic leukemia (APL) and, to a lesser extent, in core-binding factor (CBF) AML and in AML with NPM1 mutation (16–18). Those AML subtypes have canonical genetic translocations that are essential for the pathogenesis of the leukemia and are present almost uniformly in all leukemia cells and subclones. Overall, more data are available in the post HSCT setting, where MRD may be used in order to trigger interventions aimed to reduce hematological relapse risk (19, 20). In this issue, Fan et al. report on the efficacy of different immune intervention strategies in AML patients with t (8;21), according to PCR-based MRD levels after HSCT. They showed that whereas administration of IFNa appears to be the best strategy for patients with low levels of RUNX/RUNXT1 transcript, for patients with higher burden of disease, donor lymphocyte infusions (DLI) represent the most efficient immunological intervention.

In patients lacking a specific transcript, aspecific markers could be used with the same aim. In this view, Georgi et al. reported how donor/recipient chimerism assessment may effectively trigger immune intervention in most AML patients.

Another issue of MRD assessment in AML is represented by the genetic heterogeneity of leukemic cells, both within an individual patient and between different patients (20). Even if nowadays many details on the molecular complexity of AML have been discovered, it is still impossible to identify the biological characteristics of the subset of AML stem cells, which are able to cause relapse in the single patient. It is also very difficult to distinguish with conventional methods those leukemic stem cells (LSC) from the bulk of the disease (20). Furthermore, recent data suggest that, besides LSC, there are also larger subclones of more committed leukemic cells, retaining LCS-like properties, that are able to cause relapses as well (21). This may reflect one of the possible mechanisms of clonal shift in AML, explaining why at relapse immunophenotypic and genetic features may not completely correspond to the ones at diagnosis. High level of MRD after treatment may reflect the presence of larger compartments of chemo-resistant LCS or with stem cell-like properties, as suggested by Kamel et al.

Finally, in the last year with the introduction of the combination of hypomethylating agents and novel drugs such as the BCL-2 inhibitor Venetoclax, the treatment of elderly AML patients has dramatically changed (22–24). However, there are still few data about the relevance and most informative timepoints for MRD assessment with these approaches (25, 26). In this setting, the definition of MRD itself and the timepoints for its assessment could be significantly different from intensive treatment. In the present issue, Bernardi et al. provide an updated and comprehensive review of currently available data, comparing different MRD assessment methods and timepoints. In most studies, MRD clearance in patients treated with HMA + Venetoclax seems indeed to have different kinetics than in intensively treated patients, so that further studies are warranted.

In conclusion, despite significant advances in the standardization of MRD assessment in AML, there are still only few examples of MRD-driven treatment, mostly in AML with specific genetic lesions and in the post-transplant setting.

A better understanding of the biology of the disease and the widespread introduction and integration of more sensitive and specific techniques will probably increase the clinical value of MRD information, leading to more MRD-directed clinical trials in the next future.

## Author contributions

FG: conceptualization. CV: draft writing. FG, CC and AI: draft revision. All Authors approved the final version of the manuscript.

## Conflict of interest

The authors declare that the research was conducted in the absence of any commercial or financial relationships that could be construed as a potential conflict of interest.

## Publisher’s note

All claims expressed in this article are solely those of the authors and do not necessarily represent those of their affiliated organizations, or those of the publisher, the editors and the reviewers. Any product that may be evaluated in this article, or claim that may be made by its manufacturer, is not guaranteed or endorsed by the publisher.
